# Antimicrobial Activity of Diterpenes from *Viguiera arenaria* against Endodontic Bacteria

**DOI:** 10.3390/molecules160100543

**Published:** 2011-01-13

**Authors:** Tatiane C. Carvalho, Marília R. Simão, Sérgio R. Ambrósio, Niege A. J. C. Furtado, Rodrigo C. S. Veneziani, Vladimir C. G. Heleno, Fernando B. Da Costa, Brenda P. F. A. Gomes, Maria Gorete M. Souza, Erika Borges dos Reis, Carlos H. G. Martins

**Affiliations:** 1 Laboratório de Pesquisa em Microbiologia Aplicada, Núcleo de Pesquisa em Ciências Exatas e Tecnológicas, Universidade de Franca, Franca, SP, Brazil; 2 Departamento de Ciências Farmacêuticas, Faculdade de Ciências Farmacêuticas de Ribeirão Preto, Universidade de São Paulo, Ribeirão Preto, SP, Brazil; 3 Departamento de Odontologia Restauradora, Faculdade de Odontologia de Piracicaba, Universidade de Campinas, Piracicaba, SP, Brazil

**Keywords:** antimicrobial activity, endodontic bacteria, pimarane diterpenes, structural requirements, *Viguiera arenaria*

## Abstract

Six pimarane-type diterpenes isolated from *Viguiera arenaria* Baker and two semi-synthetic derivatives were evaluated *in vitro* against a panel of representative microorganisms responsible for dental root canal infections. The microdilution method was used for the determination of the minimum inhibitory concentration (MIC) and minimum bactericidal concentration (MBC) against *Porphyromonas gingivalis*, *Prevotella nigrescens*, *Prevotella intermedia*, *Prevotella buccae*, *Fusobacterium nucleatum*, *Bacteroides fragilis*, *Actinomyces naeslundii*, *Actinomyces viscosus*, *Peptostreptococcus micros*, *Enterococcus faecalis* and *Aggregatibacter actinomycetemcomitans*. The compounds *ent*-pimara-8(14),15-dien-19-oic acid, its sodium salt and *ent*-8(14),15-pimaradien-3β-ol were the most active, displaying MIC values ranging from 1 to 10 μg mL^-1^. The results also allow us to conclude that minor structural differences among these diterpenes significantly influence their antimicrobial activity, bringing new perspectives to the discovery of new chemicals for use as a complement to instrumental endodontic procedures.

## 1. Introduction 

Bacterial infections are the major cause of pulp and periradicular pathologies [1,2]. The genera *Peptostreptococcus*, *Prevotella*, *Porphyromonas*, *Fusobacterium, Bacterioides*, and *Actinomyces* are the most frequently identified as responsible for these pathologies [1,3]. During root canal treatment, mechanical instrumentation is a very important way to eliminate microorganisms [2,4]. However, this procedure alone does not result in a bacteria-free root canal system [2,4,5]. To promote bacteria eradication, the use of chemical agents has been employed [1,2,4,5]. Nevertheless, the different susceptibilities demonstrated by the oral pathogens, as well as the toxicity and allergenicity displayed by these chemicals, have made treatment very difficult [2,3,4,5,6]. This scenario illustrates the need to discover new chemicals to be used as complement to instrumental procedures.

The wide variety of chemical structures encountered in plants provides new and important leads against pharmacological targets [7,8]. Some studies have demonstrated the great importance of botanical sources, as well as their isolated compounds, for application in dentistry [3,9,10,11,12]. Recently, our research group has concentrated efforts on the discovery of new compounds from plants that display antibacterial activity against oral pathogens. We have demonstrated that some pimarane-type diterpenes isolated from a Brazilian species of *Viguiera* are able to inhibit the growth of various cariogenic bacteria, such as *Streptococcus salivarius*, *S. sobrinus*, *S. mutans*, *S. mitis*, *S. sanguinis*, and *Lactobacillus casei*, with very promising MIC values ranging from 2 to 10 μg mL^-1^ [12]. We have also pointed out that this class of diterpenes may be potentially employed in the further development of new natural anti-caries agents [12]. In view of these significant results against oral bacteria as well as the need of novel compounds that can be employed as endodontic irrigants, we have decided to investigate the effect of these natural products on a panel of representative microorganisms responsible for root canal infections.

## 2. Results and Discussion 

**Figure 1:**
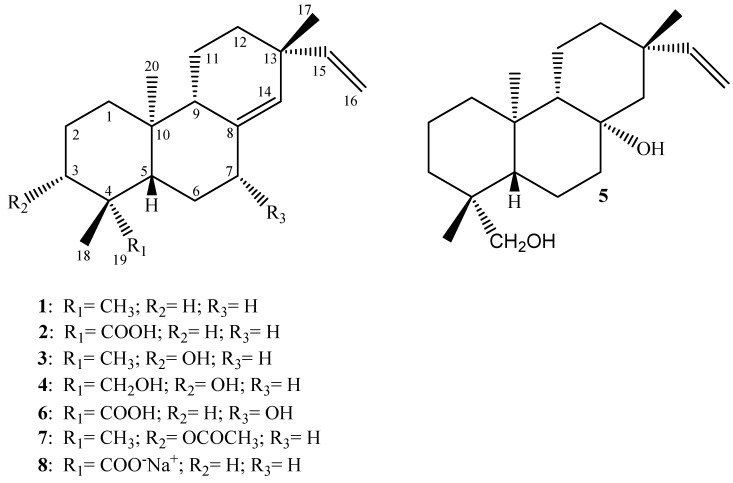
Chemical structures of the diterpenes evaluated in the present work.

The chemical structures of the diterpenes evaluated in the present work are shown in Figure 1. The spectral data of all compounds are in agreement with those previously reported in the literature: **1**, **2**, **5**, and **6** [13,14], **3** [15], **4** [16], **7** and **8** [12]. The ^1^H- and ^13^C-NMR spectral data indicate that the purity of each evaluated compound grade fell between 95-98%.

In the present work we describe the effective antimicrobial activity displayed by some pimarane-type diterpenes isolated from the dichloromethane extract of the roots of *V. arenaria* (DREVa) as well as two semi-synthetic derivatives **7** and **8** against important endodontic anaerobes by means of MIC and MBC values. Analysis of our results (Table 1) show that the compounds *ent-*pimara-8(14),15-dien-19-oic acid (PA, **2**), *ent*-8(14),15-pimaradien-3β-ol (**3**) and the sodium salt **8** of PA, were the most active metabolites, showing very promising MIC values for most of the evaluated pathogens.

It is noteworthy that these metabolites inhibited [17,18] the growth of several important ATCC and clinical isolated bacteria involved in endodontic pathologies very efficiently. However, analysis of the results achieved for *F. nucleatum* (ATCC 25586), *A. naeslundii* (clinical isolate), *A. viscosus* (clinical isolate), *E. faecalis* (ATCC 4082 and isolated) reveals that these strains are not susceptible to any of the evaluated metabolites. Table 2 shows the MBC values of compounds **2**, **3**, and **8** for the strains whose MIC values were equal to or lower than 10.0 µg mL^-1^. The MBC values for these diterpenes were equal to or 2-8 times higher than its MIC values.

According to Ríos and Recio [17] and Gibbons [18], some considerations must be borne in mind in the study of antimicrobial assays of plant extracts, essential oils, and compounds isolated from natural sources. These authors have emphasized that MIC values higher than 100.0 μg mL^-1^ for pure metabolites are evidence of poor activity. On the other hand, isolated compounds that inhibit the growth of the microorganisms at concentrations below 10.0 μg mL^-1^ are considered very promising in the search for new anti-infection agents. On the basis of these criteria and analyzing ours results (Table 1), the natural pimarane-type diterpenes **2** and **3,** as well as the semi-synthetic compound **8** can be classified as potential candidates for the discovery of new antibacterial agents against endodontic pathogens. These results, in addition to the works previously reported in the literature about the antimicrobial activity of pimarane diterpenes [12,19,20] reinforce the great importance of this class of natural products in the search for new effective antimicrobial agents for application in dentistry.

Several studies have demonstrated the significant antimicrobial activity displayed by diterpenes [12,19,21,22,23,24,25]. The mechanism(s) behind a possible antimicrobial effect of this class of natural products have not yet been elucidated. Recently, Urzúa *et al.* [26] and Wilkens *et al.* [27] suggested that these metabolites promote bacterial lysis due to their insertion into the lipophilic cell membrane and its consequent disruption. According to these authors, the structural features that promote the efficient antibacterial effect displayed by these natural compounds include a lipophilic decalin ring system, capable of insertion into a lipophilic region, and one strategically positioned hydrogen-bond-donor group (HBD; hydrophilic group), capable of interactions with the membrane phosphorylated groups. Moreover, in this study it was emphasized that a second HBD introduced in the decalin ring system led to reduction in or suppression of the activity. A careful observation of the results (Table 1) reveals that compounds **2**, **3**, and **8**, which contain only one HBD at C-3 or C-19, display MIC values much lower than that observed for diterpene **1**, which has no HBD. Also, it is noteworthy that the presence of two HBDs in the *ent*-pimaranes skeletons (compounds **4**, **5** and **6**) drastically reduces the antimicrobial activity displayed for most pathogens evaluated in the present study. Based on these considerations, our results give support to the mechanism of action suggested by Urzúa *et al*. [26] and Wilkens *et al.* [27]. 

**Figure 2:**
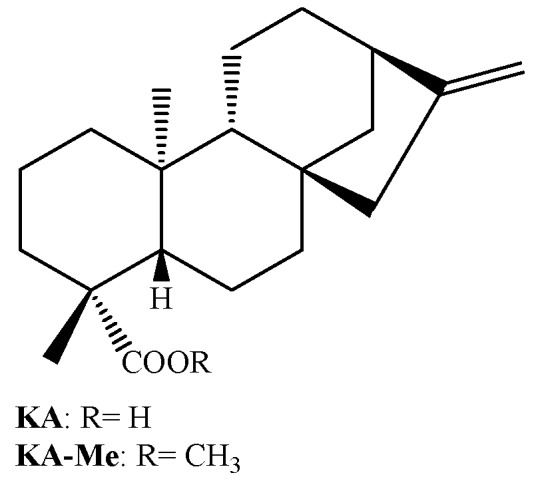
Chemical structure of kaurenoic acid (KA) and its respective methyl ester (KA-Me).

Urzúa *et al*. [26] have also demonstrated that the efficiency of hydrogen-bond-interactions between the phosphorylated groups in the membrane and the HBD in the diterpene are very important for the antibacterial activity. In this work it is demonstrated that kaurenoic acid (KA; Figure 2) is much more active than its respective methyl ester (KA-Me; Figure 2). The docking of these compounds into a model using phosphatidylcholine bilayer revealed that KA-Me is more deeply embedded into the hydrophobic region of the membrane compared with KA, presumably diminishing the capacity of the molecule to disrupt or damage the cell membrane. This observation is also in agreement with our results when the MIC values displayed by the *ent*-pimarane diterpenes **3** and **7** are compared (Table 1). For these compounds, the fact that the hydroxyl at C-3 is substituted with an acetyl group considerably diminishes its antimicrobial activities.

Also in agreement with this idea, it is remarkable that the sodium salt of PA (compound **8**), obtained by saponification of PA, exhibits improved antimicrobial activity against the tested endodontic bacteria, compared with its parent form. In fact, our research group has already observed this pattern of activity when considering PA, its sodium salt, and a panel of Gram positive bacteria [12,19]. Moreover, this rule can also be applied to the antimicrobial activity displayed by another diterpene acid (*ent*-kaurenoic acid, KA, Figure 2) against oral pathogens (unpublished results). These results suggest that obtaining salts from acidic diterpenes is a successful strategy for increasing its antimicrobial activity.

According to several authors [7,8,28], drugs derived from medicinal plants can serve not only as new drugs themselves but also as prototypes suitable for optimization through several approaches, including medicinal and semi-synthetic strategies. In summary, pimarane-type diterpenes are an important class of natural products and should be considered in the search for new dental root canal irrigants.

## 3. Experimental 

### 3.1. General

NMR spectra were recorded on a Bruker DPX 400 spectrometer (400 MHz for ^1^H and 100 MHz for ^13^C). Samples were dissolved in CDCl_3_ or CD_3_OD, with TMS as internal reference; chemical shifts are given in ppm.

### 3.2. Plant material 

*Viguiera arenaria* Baker (Asteraceae) was collected by F.B. Da Costa from the vicinity of the Washington Luís highway (km 223, 22°10 S, 47°59 W, SP, Brazil, in March 1999), and was identified by J. N. Nakajima (Universidade Federal de Uberlândia, MG, Brazil) and E. E. Schilling (University of Tennessee, TN, USA). A voucher specimen was deposited in the herbarium of the Departamento de Biologia; Faculdade de Filosofia, Ciências e Letras de Ribeirão Preto; Universidade de São Paulo; SP; Brazil (SPFR 4006).

### 3.3. Extraction and isolation

An aliquot (8.0 g) of the dichloromethane crude extract from the roots of *V. arenaria* (VaDRE) was suspended in a CH_3_OH/H_2_O solution (9:1 v/v) and partitioned with *n*-hexane and dichloromethane (DCM) (5 x 300 mL). The *n*-hexane (2.0 g) and DCM fractions (1.0 g) were fractioned using several chromatographic techniques, such as vacuum liquid chromatography, flash chromatography, and preparative thin-layer chromatography, as well as recrystallization with methanol, as previously described [29]. These procedures furnished the following compounds: *ent*-8(14),15-pimaradiene (**1**; 100.0 mg); *ent*-pimara-8(14),15-dien-19-oic acid (PA; **2**; 200.0 mg); *ent*-8(14),15-pimaradien-3β-ol (**3**; 100.0 mg); *ent*-8(14),15-pimaradiene-3β,19-diol (**4**; 20.0 mg); *ent*-15-pimarene-8β,19-diol (**5**; 5.0 mg); 7β-hydroxy-*ent*-pimara-8(14),15-dien-19-oic acid (**6**; 10.0 mg).

### 3.4. Semi-synthetic derivatives 

Compound **7** (37.0 mg) was obtained from **3** (50.0 mg) as described by Da Costa *et al.* [30]. Compound **8** (40.0 mg) was prepared from **2** (50.0 mg) according to Daló *et al.* [31]. The chemical structures of these compounds were established by ^1^H- and ^13^C-NMR spectral data analysis and comparison with literature data [12].

### 3.5. Determination of the minimal inhibitory concentration and minimal bactericidal concentration

The minimal inhibitory concentration values (MIC) and the minimal bactericidal concentration (MBC) of the pure diterpenes were determined in triplicate by using the microdilution broth method in 96-well microplates [32,33]. The tested strains were obtained from the American Type Culture Collection (ATCC) and clinical isolate. The following microorganisms were used in the present work: *Porphyromonas gingivalis* (ATCC 33277), *Porphyromonas gingivalis* (clinical isolate), *Prevotella nigrescens* (ATCC 33563), *Prevotella intermedia* (clinical isolate), *Prevotella buccae* (clinical isolate), *Fusobacterium nucleatum* (ATCC 25586), *Bacteroides fragilis* (ATCC 25285), *Actinomyces naeslundii* (ATCC 19039), *Actinomyces naeslundii* (clinical isolate),* Actinomyces viscosus* (clinical isolate), *Peptostreptococcus micros* (clinical isolate), *Enterococcus faecalis* (ATCC 4082), E*nterococcus faecalis* (clinical isolate), and *Aggregatibacter actinomycetemcomitans* (ATCC 43717). The samples were dissolved in dimethyl sulfoxide (DMSO) at 1.0 mg mL^-1^, followed by dilution in schadler broth (DIFCO, Kansas City, MO, USA) supplemented with hemin (5.0 µg mL^-1^) and vitamin K1 (10 µg mL^-1^) for anaerobic bacteria; *E. faecalis* and *A. actinomycetemcomitans* were diluted in tryptic soy broth (DIFCO) as a facultative microorganism and microaerophili, respectively; concentrations ranging from 80.0 to 0.25 μg mL^-1^ were achieved. The final DMSO content was 5% (v/v), and this solution was used as negative control. The inoculum was adjusted for each organism, to yield a cell concentration of 5 × 10^5^ colony forming units (CFU) mL^-1^, according to previously standardization by the Clinical Laboratory Standards Institute [32,33]. One inoculated well was included, to allow control of the adequacy of the broth for organism growth. One non-inoculated well, free of antimicrobial agent, was also included, to ensure medium sterility. Chlorhexidine dihydrochloride was used as positive control. The microplates (96-wells) were sealed with plastic film. The time necessary for growth was 24 hours for *E. faecalis* and *A. actinomycetemcomitans*, and 96 hours for anaerobes, incubated at 37 °C under appropriate gaseous conditions [facultative in aerobiose; microaerophili in an atmosphere of 10% CO_2_; anaerobe in an anaerobic work station (Don Whitley Scientific, Bradford, UK), in an atmosphere of 5–10% H_2_, 10% CO_2_, 80–85% N_2_]. After that, resazurin (30 μL) in aqueous solution (0.02%) was added to the microplates, to indicate microorganism viability [34]. Before the addition of resazurin and in order to determine MBC, an aliquot of the inoculum was aseptically removed from each well presenting no apparent growth, and then plated onto agar Schadler (DIFCO) supplemented with hemin (5.0 µg mL^-1^), vitamin K1 (10.0 µg mL^-1^), and sheep blood (5%) for anaerobic bacteria. *E. faecalis* and *A. actinomycetemcomitans* were plated in tryptic soy agar (DIFCO) supplemented with 5% sheep blood; the plates were incubated as previously described. Determination of the MBC values was only performed for the most active compounds (**2**, **3** and **8**), against the strains whose MIC values were very promising (equal to or lower than 10.0 µg mL^-1^) [17,18].

## 4. Conclusions 

According to several authors [7,8,28], drugs derived from medicinal plants can serve not only as new drugs themselves, but also as prototypes suitable for optimization through several approaches, including medicinal and semi-synthetic strategies. According to our rersults pimarane-type diterpenes are an important class of natural products and should be considered in the search for new dental root canal irrigants.

## Figures and Tables

**Table 1:** *In vitro* antibacterial activity (MIC) of the pimarane-type diterpenes against endodontic pathogens.

Microorganism (ATCC)	Minimum Inhibitory Concentration (μg mL^-1^)								
	1	2	3	4	5	6	7	8	Chlor
*P. gingivalis* (33277)	*	1.25	10.0	40.0				1.0	0.92
*P. gingivalis* (clinical isolate)	*	2.0	10.0	*	*	*	60.0	1.25	1.85
*P. nigrescens* (33563)	*	2.0	10.0	50.0	60.0	60.0	40.0	1.5	0.92
*P. intermedia* (clinical isolate)	*	1.0	7.5	50.0	40.0	60.0	20.0	1.75	0.92
*F. nucleatum* (25586)	*	*	*	*	*	*	*	*	1.85
*P. buccae* (clinical isolate)	*	4.0	4.0	4.0	1.5	2.25	1.75	2.25	0.92
*B. fragilis* (25285)	*	2.0	2.5	40.0	40.0	18.0	16.0	10.0	7.38
*A. naeslundii* (19039)	*	1.25	5.0	12.0	60.0	60.0	40.0	5.0	1.85
*A. naeslundii* (clinical isolate)	*	*	*	*	*	*	*	*	1.85
*A. viscosus* (clinical isolate)	*	*	*	*	*	*	*	*	3.69
*P. micros* (clinical isolate)	*	0.5	6.0	16.0				8.0	1.85
*E. faecalis* (4082)	*	*	*	*	*	*	*	*	3.69
*E. faecalis* (clinical isolate)	*	*	*	*	*	*	*	*	7.38
*A. actinomycetemcomitans* (43717)	*	4.0	10.0	*	40.0	40.0	*	1.25	7.38

* Inactive in the evaluated concentrations (MIC values higher than 80.0 μg mL^-1^); Chlorhexidine (Chlor)- positive control.

**Table 2:** Minimum bactericidal concentration for the compounds **2**, **3** and **8** against endodontic pathogens.

Microorganisms (ATCC)	Minimum Bactericidal Concentration (μg mL^-1^)			
	2	3	8	Chlor
*P. gingivalis* (33277)	10.0	10.0	8.0	0.92
*P. gingivalis* (clinical isolate)	10.0	40.0	8.0	1.85
*P. nigrescens* (33563)	2.0	40.0	1.75	0.92
*P. intermedia* (clinical isolate)	1.0	40.0	2.0	0.92
*P. buccae* (clinical isolate)	4.0	14.0	5.0	0.92
*B. fragilis* (25285)	2.0	2.5	14.0	7.38
*A. naeslundii* (19039)	10.0	5.0	5.0	1.85
*P. micros* (clinical isolate)	1.0	6.0	12.0	1.85
*A. actinomycetemcomitans* (43717)	4.0	60.0	1.75	7.38

Chlorhexidine (Chlor) - positive control; DMSO 5% water solution (negative control) did not affect the growth of the microorganisms.
